# Intratumor Heterogeneity Predicts Prognosis in Lepidic Predominant Lung Adenocarcinoma

**DOI:** 10.1111/1759-7714.15536

**Published:** 2025-01-14

**Authors:** Benedikt Niedermaier, Michael Allgäuer, Thomas Muley, Marc A. Schneider, Martin E. Eichhorn, Hauke Winter, Laura V. Klotz

**Affiliations:** ^1^ Department of Thoracic Surgery, Thoraxklinik Heidelberg University Hospital Heidelberg Germany; ^2^ Translational Lung Research Center Heidelberg (TLRC‐H), Member of the German Center for Lung Research (DZL) Heidelberg Germany; ^3^ Institute of Pathology Heidelberg University Hospital Heidelberg Germany; ^4^ Translational Research Unit, Thoraxklinik Heidelberg University Hospital Heidelberg Germany

**Keywords:** histology, lepidic, lung cancer, prognosis, survival

## Abstract

**Objective:**

Among the different subtypes of invasive lung adenocarcinoma, lepidic predominant adenocarcinoma (LPA) has been recognized as the lowest‐risk subtype with good prognosis. The aim of this study is to provide insight into the heterogeneity within LPA tumors and to better understand the influence of other sub‐histologies on survival outcome.

**Methods:**

Overall, 75 consecutive patients with LPA in pathologic stage I (TNM 8th edition) who underwent resection between 2010 and 2022 were included into this retrospective, single center analysis. The proportions of different growth patterns were reported in 5% increments according to the WHO classification.

**Results:**

All tumors exhibited a predominantly lepidic growth pattern (median proportion 70%, IQR 60%–85%). The invasive component included acinar (*n* = 66, 88%), papillary (*n* = 41, 55%), micropapillary (*n* = 14, 19%), and solid growth patterns (*n* = 4, 5%), with most tumors exhibiting more than one invasive growth pattern. The presence of high‐risk growth, that is, micropapillary and solid, was associated with higher T stage (*r* = 0.423, *p* = 0.0002). A classification of patients as lepidic/high‐risk or lepidic/low‐risk based on the presence of micropapillary and solid growth patterns resulted in a significantly worse disease‐free survival (*p* = 0.0169, 5‐year DFS: lepidic/high‐risk 73% vs. lepidic/low‐risk: 95%) for the lepidic/high‐risk group, while the groups did not differ in age, gender, smoking status, or extent of resection.

**Conclusion:**

Patients with stage I LPA exhibit considerable intratumor heterogeneity regarding growth patterns, which can be used for prognostic stratification. The occurrence of micropapillary and solid growth patterns in LPA is associated with poorer disease‐free survival.

## Graphical abstract

We report the histomorphological composition of lepidic‐predominant lung adenocarcinoma and found considerable heterogeneity. The occurrence of solid and micropapillary growth was associated with poorer disease‐free survival and higher T‐stage. Prognostic stratification of LPA patients should account for the heterogeneity in growth pattern composition.

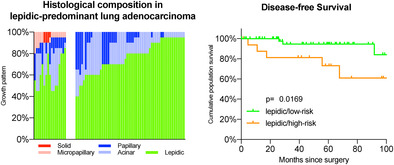

## 1 Introduction

Lung adenocarcinoma (LUAD) is the most prevalent histologic type of primary lung cancer, which remains the most common cause of cancer‐related death globally [1]. However, LUAD is widely recognized as a heterogeneous group of cancers with considerable differences in histomorphological appearance and prognosis [2, 3]. The 2021 WHO classification of lung cancer recognizes six major growth patterns of nonmucinous lung adenocarcinoma: lepidic, acinar, papillary, micropapillary, solid, and complex glandular [4]. Most LUAD present as heterogeneous tumors consisting of multiple growth patterns, and prognosis has been linked to the proportion of each histopathologic component in numerous studies [5, 6, 7]. Among the different subtypes based on predominant growth pattern, lepidic‐predominant adenocarcinoma (LPA) has the most favorable prognosis, whereas micropapillary‐predominant and solid‐predominant adenocarcinoma have less favorable outcomes [2, 3]. The lepidic growth pattern is characterized by growth of neoplastic cells along preexisting alveolar structures without signs of stromal, vascular, or pleural invasion [8]. Lepidic growth therefore refers to the noninvasive component within an adenocarcinoma, in contrast to the other, invasive growth patterns [4, 9]. The term LPA is recommended for any invasive adenocarcinoma with a largest percentage of lepidic growth; additionally, nonpredominant invasive components should be reported in 5% increments [4, 8].

The concept of progression from lepidic precursor lesions to lepidic‐predominant adenocarcinoma and finally to an adenocarcinoma with predominant invasive pattern has not been proven but is backed by a growing amount of evidence [10, 11, 12]. Recent data show a gradual decrease in survival with advancing lesions [10, 13]. In this context, heterogeneity within LPA is thought to reflect a chronological progression, but direct evidence is lacking.

Although LPA has the best prognosis of the different LUAD tumors, the characteristics of this group of patients remain largely unknown. In this exploratory analysis, we therefore aim to analyze the histomorphological and clinical features of patients with LPA to assess the characteristics of invasive growth and the impact on disease prognosis.

## 2 Methods

### 2.1 Patients

**FIGURE 1:**
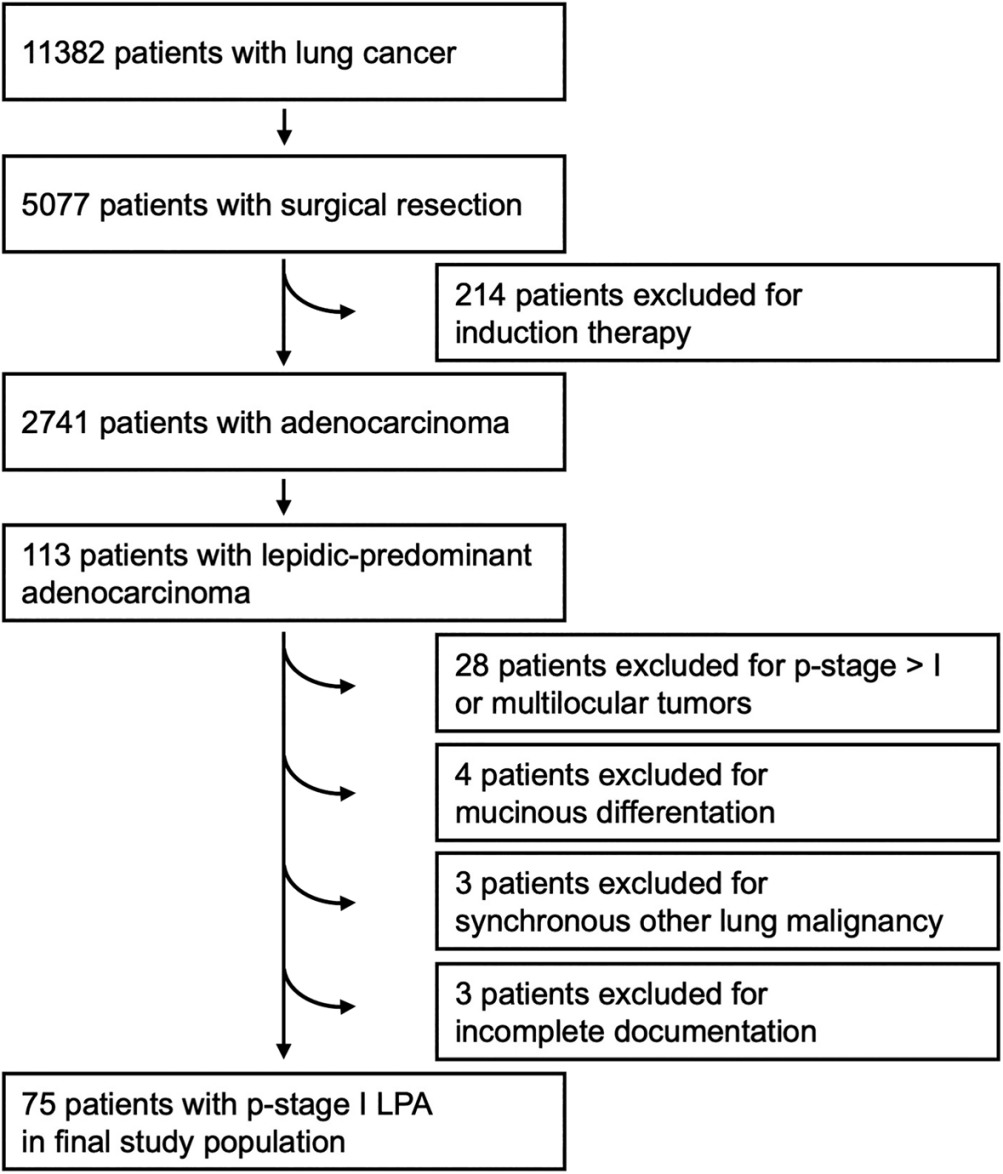
Flow chart for the selection of patients into the study cohort from a single center between 2010 and 2022.

This retrospective study was approved by the ethics committee Heidelberg (S‐174/2019). Hospital records from 2010 to 2022 were retrospectively reviewed to identify patients who underwent resection of LPA in pathological TNM stage I (see Figure 1 for a flow chart for the selection of patients to be included). Patients with multilocular tumors, tumors with mucinous differentiation, carcinoma in situ, synchronous other lung malignancy, and predominance of other growth patterns were excluded. Clinical and pathological data such as age, gender, tumor location, histology, surgical procedure, and *p*‐TNM stage were extracted from the hospital database and used for further analysis. TNM descriptors were categorized according to the 8th edition of the AJCC TNM staging system, with reclassification of patients originally staged according to the 7th edition where appropriate. All patients had oncological complete (R0) resection.

### 2.2 Histopathologic Analysis

The proportions of different growth patterns were delineated in 5% increments according to the WHO classification [4]. To aid distinction of different growth patterns, for example, lepidic and papillary growth, elastic stains of two tumor‐bearing FFPE blocks of each resected lung cancer specimen were performed as part of the routine work‐up. Histopathological assessment was carried out by a dedicated team of only four pathologists routinely seeing all thoracic cases. While this team was composed of different members over the course of the study period due to the academic nature of our practice, consistency was ensured by proper training and having one main pulmonary pathologist at any given time. Of note, our group was early on and prominently involved in validation [3] and also interobserver studies [14, 15] of the WHO grading system, setting the ground for reproducible assessment of growth patterns in our clinical care. Difficult cases were discussed if needed. There was no systematic retrospective reevaluation of cases for this study. The predominant pattern was defined as the pattern with the largest percentage, even if below 50%. Patients with a predominance of lepidic growth were considered for inclusion in the study population as outlined in Figure 1.

### 2.3 Follow‐Up

Postoperative follow‐up was routinely performed every 3 months in the first year after surgery, every 6 months until the fifth postoperative year and annually thereafter in accordance with the applicable guidelines. Routine examination included a medical history review, physical examination, chest x‐ray, and contrast‐enhanced computed tomography every 6 months. The images were interpreted by radiologists specializing in oncological thoracic imaging. Histological evidence of lung adenocarcinoma was considered disease recurrence, or radiological detection of progressive nodules in local relation to the primary disease. Local recurrence was defined as disease recurrence at the surgical margin, ipsilateral hemithorax, or ipsilateral mediastinal lymph nodes. All other locations, including the contralateral lung, were defined as distant recurrence. Patients were censored at the onset of any other primary or secondary lung malignancy.

### 2.4 Statistical Analyses

Statistical analyses were performed using Prism (version 8.2.1, Graphpad Software Inc., Boston, USA). Demographic, clinical, and pathologic characteristics were reported in descriptive analysis presenting continuous variables as median with interquartile range, and categorical variables as number and percentage. Patients with LPA including high‐risk growth patterns (solid and micropapillary) were compared to LPA with only acinar and papillary invasive growth patterns regarding clinical and pathological data and outcomes. The Wilcoxon test was used to compare group differences in continuous variables, and the chi‐squared test was used for categorical variables. Disease‐free survival, overall survival and cancer‐specific survival were analyzed using the Kaplan–Meier method with the log‐rank (Mantel‐Cox) test. Disease‐free survival was defined as the time between surgical intervention and recurrence of the disease, overall survival as the time between surgical intervention and death, and cancer‐specific survival as the time between surgical intervention and tumor‐related death. *p* values less than 0.05 were considered significant.

## 3 Results

**FIGURE 2:**
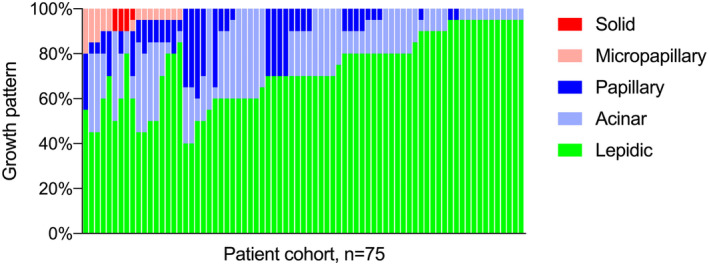
Intratumor heterogeneity in patients with lepidic predominant adenocarcinoma. The proportion of subtypes is indicated for each individual patient.

**FIGURE 3:**
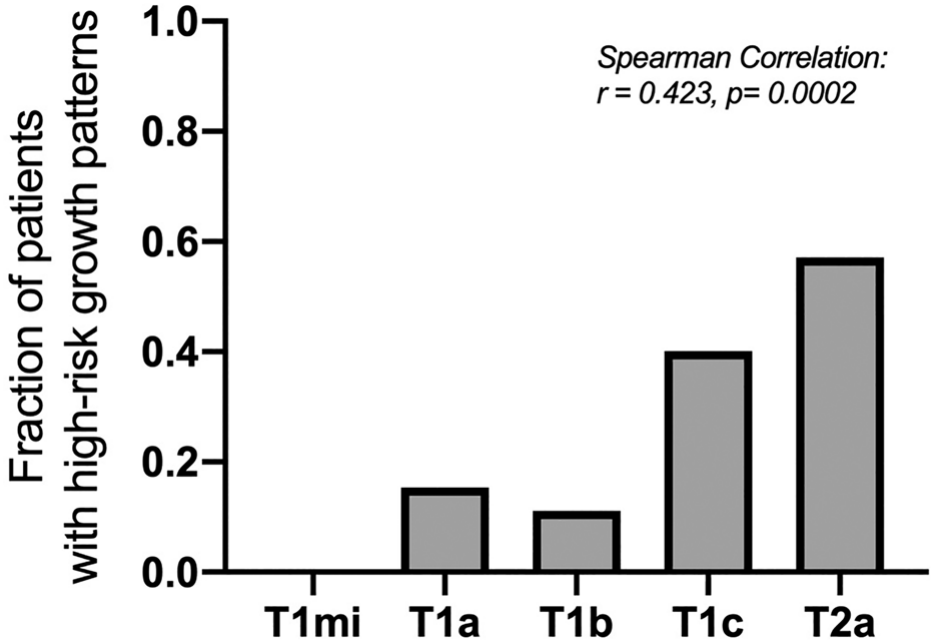
A higher T‐stage is associated with the occurrence of high‐risk growth patterns in LPA. The graph shows the proportion of patients with solid or micropapillary growth regions in relation to the T‐descriptor. Spearman nonparametric correlation is given with *r* = 0.423, *p* = 0.0002.

This retrospective, single‐center study included 75 consecutive patients with pathological stage I LPA who underwent curative‐intent surgery (Table 1). The average age of the included patients was 69 years (IQR 61–75). There were 33 male patients (44%) and 42 (56%) female patients. Lobectomy was performed in 75% of patients and sub‐lobar resection in 25%. All patients had oncological complete resection (R0). Histologic evaluation revealed a marked heterogeneity of invasive growth patterns in addition to the predominant lepidic component (Figure 2). The median proportion of lepidic growth within the entire tumor was 70% (IQR 60%–85%), the invasive component included acinar (*n* = 66, 88%), papillary (*n* = 41, 55%), micropapillary (*n* = 14, 19%), and solid subtypes (*n* = 4, 5%), with most tumors exhibiting more than one invasive growth pattern. High‐risk histopathological growth patterns such as micropapillary and solid growth, which are associated with a poorer prognosis, were found in 17 of the patients (23%). Furthermore, the presence of micropapillary or solid growth was associated with higher T stage (Figure 3; *r* = 0.423, *p* = 0.0002).

**TABLE 1:** Demographic and pathologic characteristics of the study population.

	Total	Lepidic/high‐risk	Lepidic/low‐risk	*p*
Patients, *n*	75	17	58	
Follow Up, months (median + IQR)	60 (26–82)	62 (40–93)	56 (24–79)	0.2002
Age, years	69 (61–75)	69 (61–75)	69 (61–75)	0.9750
Gender				0.7726
Male	33 (44%)	8 (47%)	25 (43%)	
Female	42 (56%)	9 (53%)	33 (57%)	
Smoking history				0.5834
Never	26 (35%)	6 (35%)	20 (34%)	
Former	37 (49%)	7 (41%)	30 (52%)	
Current	12 (16%)	4 (24%)	8 (14%)	
Tumor location				0.8619
Right upper lobe	31 (41%)	7 (41%)	24 (41%)	
Right middle lobe	1 (1%)	0 (0%)	1 (2%)	
Right lower lobe	16 (21%)	4 (24%)	12 (21%)	
Left upper lobe	25 (33%)	5 (29%)	20 (34%)	
Left lower lobe	2 (3%)	1 (6%)	1 (2%)	
T stage, *n* (%)				0.0020**
T1mi	11 (15%)	0 (0%)	11 (14%)	
T1a	13 (17%)	2 (12%)	11 (14%)	
T1b	27 (36%)	3 (18%)	24 (41%)	
T1c	10 (13%)	4 (23%)	6 (10%)	
T2a	14 (19%)	8 (47%)	6 (10%)	
Surgery				0.1435
Lobectomy	56 (75%)	15 (88%)	41 (71%)	
Sub‐lobar resection	19 (25%)	2 (12%)	17 (29%)	
Margins				—
R0	75 (100%)	17 (100%)	58 (100%)	
R1	0 (0%)	0 (0%)	0 (0%)	
Recurrence, *n* (%)				0.6733
Local	6 (8%)	4 (24%)	2 (3%)	
Distant	2 (3%)	1 (6%)	1 (2%)	

*Note:* *p* values show the result of the Chi‐Square test for categorical variables and the Mann–Whitney test for continuous variables comparing the Lepidic/high‐risk and Lepidic/low‐risk group. Bold indicates significant values.

**FIGURE 4:**
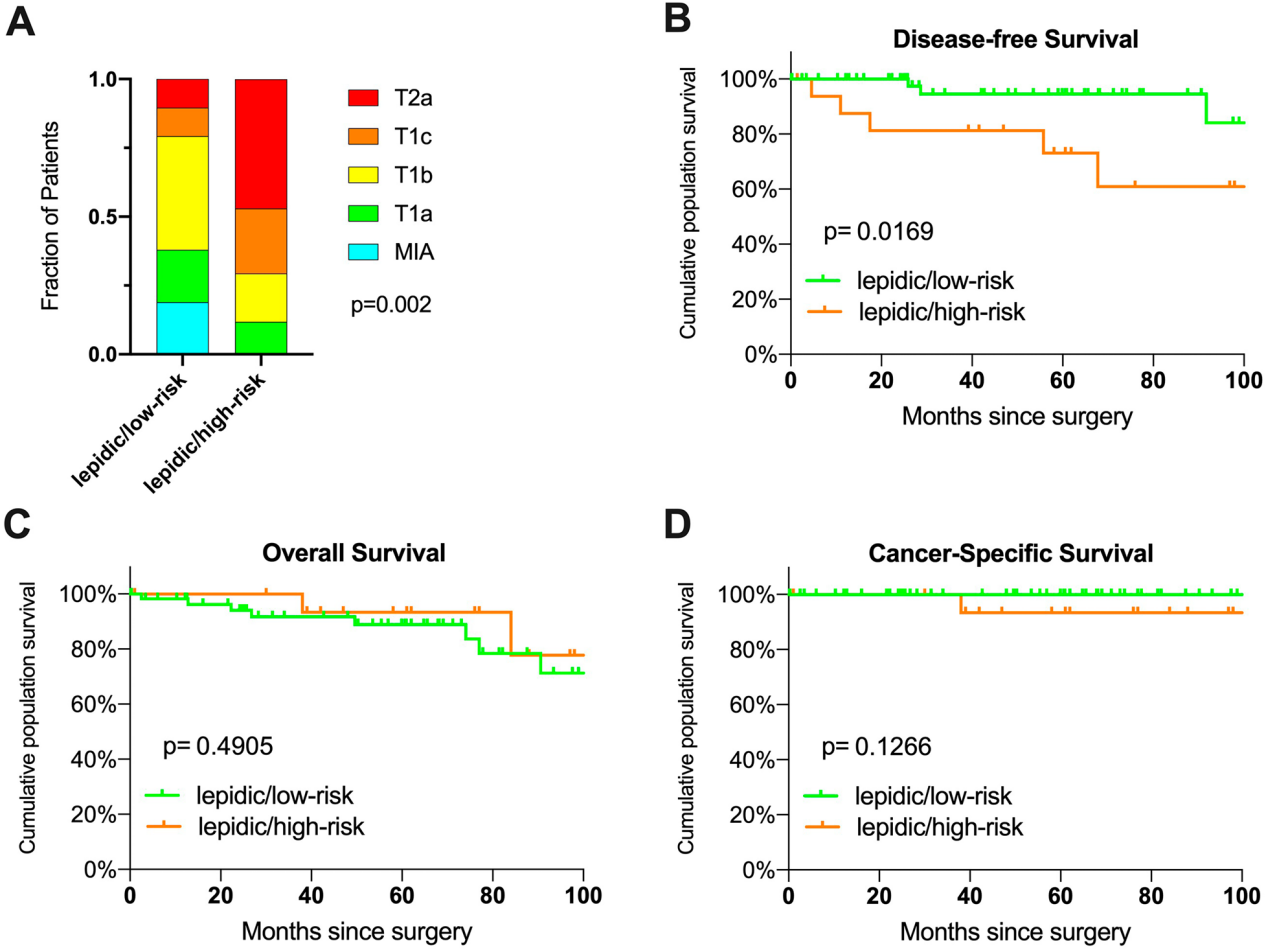
Prognosis of patients with LPA including high‐risk subtypes compared to LPA with only acinar and papillary invasive growth. (A) T‐stage distribution differs significantly between the groups (Chi‐Square test, *p* = 0.002). (B) Disease‐free survival. (C) Overall survival. (D) Cancer‐specific survival. Survival was estimated by the Kaplan–Meier method with differences analyzed using the Log‐rank (Mantel‐Cox) test assuming significance for *p* < 0.05.

The median follow‐up time of the patients was 60 months (IQR 26–82 months). Eight patients (10.7%) developed disease recurrence during the follow‐up interval. Patients with LPA including high‐risk subtypes were compared to LPA with acinar and papillary invasive growth regarding long‐term prognosis (Figure 4). The groups did not differ in age, gender, smoking status, or extent of surgical resection. The percentage of tumors with advanced T‐stages was higher in the high‐risk cohort (Figure 4A). The presence of high‐risk subtypes was associated with significantly shorter disease‐free survival (Figure 4B; *p* = 0.0169, log‐rank test. 5‐year DFS: lepidic/high‐risk 73% vs. lepidic/low‐risk: 95%). No differences were observed in overall survival or tumor‐specific survival (Figure 4C,D).

## 4 Discussion

With the implementation of lung cancer screening programs in America and many European countries, a growing number of lepidic lesions are being detected [9, 16, 17]. Initial concerns regarding overdiagnosis of otherwise indolent cancers have been invalidated by the observation that the vast majority of these lesions are classified as invasive lepidic predominant adenocarcinoma [9, 13, 16]. This development has raised new questions regarding the clinical management of LPA and the underlying tumor biology.

In the present study, a detailed analysis of patients with pathologic stage I LPA was performed. Within the study population, we found considerable heterogeneity in terms of the histomorphological growth patterns present in addition to the predominant lepidic component.

We could demonstrate that a classification of two patient groups (high‐risk/low‐risk) on the basis of the presence of micropapillary and solid tumor parts resulted in a significantly different DFS of the patients. This observation seems highly plausible in light of previous studies reporting similar outcomes in nonlepidic LUAD [18, 19, 20, 21]. Ito and colleagues found the second most common growth pattern to be of prognostic relevance in acinar and papillary predominant adenocarcinoma, reporting worse OS and DFS for tumors with the occurrence of high‐risk subtypes [18]. Kim and colleagues also identified the presence of micropapillary or solid growth as a poor prognostic factor in acinar and papillary predominant LUAD [19]. On the other hand, the proportion of lepidic growth in acinar‐predominant adenocarcinoma has repeatedly been associated with favorable prognosis [13, 18, 21]. Our study is in line with these observations while focusing on the heterogeneity in LPA, which also carries prognostic significance. These results support the recently proposed grading system by Moreira and colleagues, who argued that any tumor with 20% or more of high‐risk patterns should be classified as poorly differentiated because these tumors behave in a more aggressive fashion [2]. Similarly, in this cohort of patients with LPA, the occurrence of high‐risk patterns even at 5%–15% was associated with a decreased DFS.

The extent of surgical therapy is controversially discussed due to the comparatively favorable prognosis of patients with LPA [22, 23]. Based on the good results of the CALGB trial and the JCOG1211 trial, sub‐lobar resection is increasingly favored and the necessity of systematic mediastinal and hilar lymphadenectomy is questioned [24, 25, 26]. However, our exploratory data suggests that LPA cannot simply be regarded as a uniform group of tumors, but estimation of prognosis and therefore therapy decisions might need to account for heterogeneity in growth pattern composition. Further studies are needed to evaluate whether the indication for different surgical approaches should depend on the presence of poor histological subtypes, and evaluate the efficacy of preoperative diagnostics that are a prerequisite for such considerations.

Previous reports identified several demographic characteristics of patients with LPA, including a higher proportion of females and never‐smokers [13, 27]. While our study design was not set for comparison between LUAD with other predominant subtypes and LPA, the proportion of females (56%) does stand out in relation to the generally male‐predominant population of lung cancer patients [1].

This study is limited by its single‐center retrospective design. Although all consecutive patients with LPA histology were included and subtypes were unknown at the time of treatment, a possible selection bias cannot be ruled out. The cohort of *p*‐stage I LPA had a good prognosis, so even with a primary outcome of RFS, the number of events was small and the number of patients was relatively limited due to the rarity of LPA in the adenocarcinoma patient population. Therefore, statistical power might be improved and does not yet allow a definitive evaluation including clinical parameters such as T‐stage. Determining the independent impact of T‐stage and the presence of high‐risk subtypes on outcomes in a multivariate analysis was not possible due to the limited sample size and low incidence of recurrence. In addition, age and comorbidities may obscure the impact of LPA on prognosis due to the low aggressiveness and indolent course of LPA.

Nevertheless, this exploratory analysis sheds light on a topic of growing relevance in daily practice that urgently requires further investigation. Previous research on lepidic lesions has often included a broad spectrum of disease, that is, in situ lesions (Tis), multilocular tumors, mucinous differentiation, and minimally invasive and invasive lepidic adenocarcinoma (LPA), resulting in heterogeneous results and limited comparability due to different group compositions. We included only histologically confirmed minimally invasive and invasive LPA at pathologic stage I to ensure a transparent analysis of a homogeneous patient series and therefore accepted a limited sample size.

In conclusion, the occurrence of invasive subtypes allows LPA to be sub‐classified with prognostic significance for affected patients. Our observation suggests that the estimation of malignant potential and risk of recurrence should not only rely on the predominant lepidic component, but account for the heterogeneity within LPA. Further studies with larger cohorts are needed to validate our observations and to elucidate the underlying mechanisms involved in transition from lepidic to invasive growth patterns and their impact on tumor progression.

## Author Contributions

**Benedikt Niedermaier:** formal analysis, investigation, writing – original draft, writing – review and editing. **Michael Allgäuer:** investigation, writing – review and editing. **Thomas Muley:** resources, data curation, writing – review and editing. **Marc A. Schneider:** resources, data curation, writing – review and editing. **Martin E. Eichhorn:** writing – review and editing. **Hauke Winter:** conceptualization, writing – review and editing, supervision. **Laura V. Klotz:** conceptualization, writing – review and editing.

## Ethics Statement

The study was approved by the ethics committee of Heidelberg University under S174/2019.

## Conflicts of Interest

The authors declare no conflicts of interest.

## Data Availability

The data underlying this article will be shared on reasonable request to the corresponding author.
